# The Connection between MiR-122 and Lymphocytes in Patients Receiving Treatment for Chronic Hepatitis B Virus Infection

**DOI:** 10.3390/microorganisms11112731

**Published:** 2023-11-08

**Authors:** Marina Manea, Dimitri Apostol, Ileana Constantinescu

**Affiliations:** 1 Immunology and Transplant Immunology, University of Medicine and Pharmacy “Carol Davila”, 020021 Bucharest, Romania; 2 Centre of Immunogenetics and Virology, Fundeni Clinical Institute, 022328 Bucharest, Romania

**Keywords:** hepatitis B virus, miR-122, lymphocyte, HBV (hepatitis B virus) treatment

## Abstract

New molecular predictors for the response to treatment in HBV (hepatitis B virus) infection are assessed. Among them is miR-122. Our article searches the connection between miR-122 and the counts of lymphocytes in chronic HBV patients receiving treatment. We included the sera of 38 Romanian subjects with chronic HBV infection (20 receiving treatment and 18 not receiving treatment) and 5 healthy controls. The expression of miR-122 was determined using RT-PCR (real-time PCR) and a 2^−ΔΔCT^ method. Two systematic analyses were also performed on databases (PUBMED, Web of Science, and Science Direct), eliminating systematic reviews, editorials, letters to editors, meta-analyses, reviews, conference proceedings, or pre-print manuscripts. We included human-based articles following the PRISMA criteria and the Newcastle Ottawa Assessment Scale for Case–Control and Cohort studies. R 4.2.2 was used for statistics, and MIENTURNET and STRING were used for the bioinformatic analysis. Our results showed a link between the variations in the expression of miR-122 and the counts of lymphocytes in HBV Romanian patients receiving therapy. Treatment influenced miR-122 and the lymphocyte numbers. This is the first study with these results, and it may lead to a new perspective on the inter-relationships between microRNAs and therapy in HBV patients.

## 1. Introduction

Although vaccines and treatments have been largely implemented and are under continuous development, viral hepatitis B is still being diagnosed annually. According to the World Health Organisation (WHO), people die from viral hepatitis at a rate of 3000 persons per day, and the infection rate with HBV (hepatitis B virus) or HCV (hepatitis C virus) is 8000 new infections per day [1]. Efforts have been made to follow the 2030 elimination target for viral hepatitis, but improvements in both the diagnostic and treatment programs are needed [2]. The latest reports show that North Americans have not yet improved the survival rates of citizens suffering from chronic HBV. The development of surveillance strategies and new evaluation biomarkers are the main areas that need further attention [3]. Chronic HBV infection affects almost 6% of the adult population in some African and Asian countries. The major concern related to these infection rates is related to the liver complications caused by HBV, such as hepatocellular carcinoma (HCC). Treatment options are considered effective in reducing the rate of these complications, but no cure has yet been found [4].

HBV enters the hepatocyte through endocytosis after a viral fusion mechanism mediated by either sodium taurocholate co-transporting polypeptide (NTCP) or by large HBsAg (L-HBsAg). The following viral replication process consists of a series of steps, which include the formation of covalently closed circular DNA (cccDNA), the transcription phase, and the release of the newly formed virions. HBV-DNA is transcribed into a pregenomic RNA (pgRNA) and four messenger RNAs (mRNAs) with different lengths, which, in turn, lead to the translation of the main viral proteins: HBcAg, HBsAg, HBeAg, and HBx [5]. The pathogenic effects of HBV are related to the persistence of this virus in the host. This continuously hidden presence of the virus in the human body is achieved by the evasion of defence immune mechanisms and by the activation of a series of molecular pathways. HBsAg and HBx play a substantial role in promoting carcinogenesis [6]. HBx also interacts with various molecular pathways such as NF-kB (nuclear factor kappa B), STAT (signal transducer and activator of transcription), and ERK (extracellular signal-regulated kinase), using small noncoding molecules such as microRNAs [7]. Essentially, microRNAs are noncoding, small molecules that are processed from their initial transcripts using enzymes like Drosha and Dicer. Their main role is a regulatory one over messenger RNAs (mRNAs), and they are thought to interfere with many processes including viral replication, anti-viral immune activities, fibrotic mechanisms, cellular growth, cellular proliferation, and apoptosis [8]. Overall, HBx is important in promoting viral replication, in stimulating the production of proto-oncogenes, and in maintaining the inflammatory environment, which are contributors to a hepatocyte’s damage [7]. Small molecular interactions, mediated by miR-122, miR-29a, miR-143, miR-101, miR-132, miR-148a, miR-152, let-7, or miR-16, were studied by several scientists as key connectors between the cellular activity of HBx and carcinogenesis [9].

Chronic HBV infection is believed to affect the levels of CD4+ T lymphocytes and the quality of the response of the CD8+ T lymphocytes, thus leading to low immune activity and the persistence of the virus. On the other hand, natural killer (NK) cells and dendritic cells have an impaired function during the progression of the chronic HBV infection. This causes their apoptosis [6]. The variations in number and functionality of the immune cells are determined by molecular interactions that trigger the release of cytokines. The most affected immune cells in chronic HBV infection are the lymphocytes [10]. Therefore, these cells are being studied in future immunologic therapies meant to cure chronic HBV infection [6]. 

All the currently existing guidelines agree on classifying chronic HBV infection into five categories of illness. Although the terminology of each phase is different amongst the various guidelines, all of them are based on the quantification of some viral proteins (HBsAg and HBeAg), the level of HBV-DNA, the level of alanine aminotransferase (ALT), and the histological assessment of the liver. Some of these guidelines are based upon the intensity of the viral replication, while others include the level of liver damage. [11]. Every phase of the chronic HBV infection is characterised by alterations in the numbers and functionalities of several lymphocytes [10]. On the other hand, microRNAs interfere in the progression of chronic HBV infection and influence the development of liver cancer. MiR-122 is one of the most studied microRNAs related to chronic HBV. Some authors show that the expression of miR-122 is stimulated, while others mark a downregulation of this microRNA in chronic HBV patients. Nevertheless, researchers also studied some other microRNAs during the progression of the HBV infection, such as miR-223, miR-155, miR-29a, and miR-125a [12]. The exact influencers of the activities of microRNAs have not been yet discovered. 

In a recent study, 45 Iranian subjects carrying HBV were compared to 15 healthy subjects. The researchers found that the expression of miR-122 is different in the patient lot from the other lot. However, miR-122 detected in human serum could not be used for the intergroup differences between patients [13]. On the other hand, in a study performed on Chinese patients, the expression of miR-122 determined from plasma samples was significantly different between active HBV subjects and simple viral carriers [14]. Scholars have even indicated that genetic mutations could affect the expression of miR-122 and prevent the chronic evolution of the HBV infection. The same mutations seemed to prevent HCC related to HBV [15]. The problem of carcinogenesis related to chronic HBV was also addressed in various articles. A recent meta-analysis summarises the obtained results and concludes that the expression of miR-122 is a potential reliable biomarker in the detection of HCC related to HBV [16].

Currently, there are two main treatment options for chronic HBV: an interferon-based therapy and a treatment with nucleotide/nucleoside analogues (NUCs) [17]. HBV treatment during the chronic infection is prescribed after the assessment of liver damage and after HBV-DNA quantification (an expensive test in low-economy countries). Some other criteria (such as the family background of HCC) are also involved in establishing the proper moment for therapy [18,19]. NUC therapy is usually the common option because of its advantages regarding the decrease of viremia and because of the long-term effects in comparison with the pegylated interferon (pegIFN) treatment. However, resistance to NUC therapy is possible [18]. The proper moment for ceasing treatment in chronic HBV patients is also a subject of debate because current recommendations differ [20]. NUC treatments are also considered expensive in some low-income countries [21], where the patient’s adherence and access to therapy are not optimal [1,2,21]. Moreover, some authors find that current guidelines do not necessarily indicate the correct starting point for the treatment of chronic HBV. However, untreated patients are sometimes at risk for complications [22]. Other researchers emphasise that current therapies are not a cure for HBV infection, even on a functional basis, and that new treatment options should be considered [12,23,24]. 

This context shows that treating chronic HBV patients can sometimes be challenging and that the main problems are related to the response to treatment or to the adherence to therapy. The best cessation moment for each treatment is also a current concern. Therefore, biomarkers that answer all these requirements are currently being investigated. Some authors have focused their attention on biochemical markers (such as the levels of transaminases and glucose), and viremia as early indicators for response to treatment in chronic HBV patients [25]. A series of studies have focused on the levels of various immune cells as potential predictors of the treatment’s efficacy [10]. Others emphasise the complex role of microRNAs in the viral replication process and their connections with both viral proteins and the clinical stage of the HBV infection. MicroRNAs are also thought to have a high potential in the evaluation of the response to treatment [26]. MiR-122 is a potential biomarker for the assessment of chronic HBV [26,27], together with viremia and the levels of HBsAg [27]. This makes this microRNA an ideal candidate for the assessment of the response to HBV therapy. 

On the other hand, recent studies have shown that the loss of HBsAg in chronic HBV patients receiving treatment is related to their viral genotype [28]. In Romania, the dominant viral genotypes are A and D [29], which are different from the dominant genotypes found in Asian subjects (who are mostly involved in HBV studies) [30]. Since microRNAs and lymphocytes are also involved in treatment response, the differences in viral genotypes might affect them as well. 

Our article was designed as a multiphase study, and it intended to retrieve the known molecular pathways related to the HBV treatment response and the expression of microRNAs. Following recent discoveries, we focused on the connection between miR-122 and the levels of lymphocytes as a molecular interplay influenced by therapy in Romanian patients. Although the involvement of miR-122 was already documented in HBV infection, our article sets the attention, for the first time, on another aspect, that is, the connection between miR-122 and the levels of lymphocytes in Romanian chronic HBV patients receiving treatment. 

The novelty of this article can be observed. This is, to our knowledge, the first article with the above-mentioned purposes, based on such an amount of documentation. Our article contains the first pilot study which sets a trend related to the connection between miR-122 and the levels of lymphocytes in HBV positive Romanian patients receiving treatment. Such subjects were never assessed from this point of view, and this is important because the dominant viral genotype of Romanian patients is generally different from the HBV genotypes of other studied populations. These differences might be reflected in the molecular interactions and the response to treatment.

## 2. Materials and Methods

### 2.1. The Article’s Main Parts

**Figure 1:**
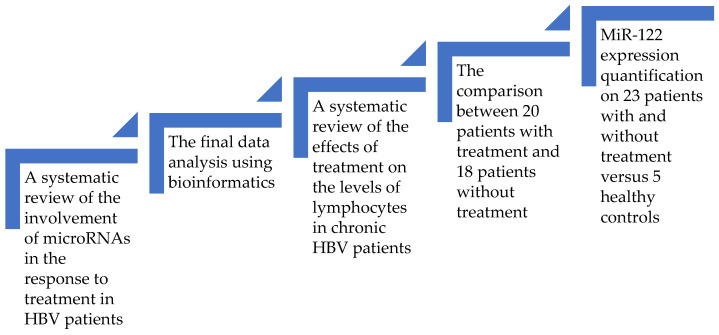
The design of this study.

The first part of the article consists of a systematic analysis of the microRNAs related to the response to treatment in HBV patients. Then, using a bioinformatic approach, we tried to retrieve the molecular pathways, which included the majority of the previously found microRNAs. Based on our discoveries, we then assessed the impact of pegIFN or NUC therapy on the levels of the lymphocytes. The final step was a clinical study performed on a lot of treated versus untreated patients. The goal was to prove that treatment influences the levels of lymphocytes in Romanian HBV patients. Then, we randomly selected 23 patients (some of them were receiving treatment and others were not), and we added 5 healthy controls to evaluate the connection between therapy and the variations in the expression of miR-122 and the levels of lymphocytes. The general design of this multiphase study is depicted in Figure 1. Our general aim was to overview the current knowledge about microRNAs in the treatment of chronic HBV subjects and to connect it with our findings.

### 2.2. MicroRNAs Related to Treatment in Chronic HBV Patients

The first systematic analysis comprised three databases (PUBMED, Web of Science, and Science Direct). The main goal was to retrieve the microRNAs related to the response to NUC or pegIFN treatment in chronic HBV patients. We inserted in the search motor various words (and their derivatives): “nucleoside”, “nucleotide”, “pegIFN”, “treatment”, “Chronic hepatitis B (or CHB)”, “VHB” (viral hepatitis B), “HBV”, “miRNA”, and “microRNA”. We obtained 4642 records, but we only included the already published articles. All systematic reviews, editorials, letters to editors, meta-analyses, reviews, conference proceedings, or pre-print manuscripts were not taken into consideration. The retrieved articles were then carefully assessed, and we eliminated those that were not directly connected to our goal. The time frame accepted as a publishing date for the included articles was from the beginning of the time to 26 October 2023. All articles without freely available data were excluded, together with those performed on non-human subjects. The overviewed data was related to the main characteristics of the articles (the date of publication, the location of the study, the type of study), to some of the details concerning the subjects (the sample size, the groups of patients included in the studies), and to a series of information about the microRNAs involved (the detection method, the expression pattern observed). All articles were archived using ZOTERO (http://www.zotero.org, accessed on 23 February 2023) [31]. This software was also used for the removal of duplicates. This entire work was elaborated after the PRISMA criteria [32]. The retrieved studies were evaluated using the Newcastle Ottawa Assessment Scale for Case–Control and Cohort studies [33]. The entire process was performed by the first author; the following author checked the results. The last author approved the final work. 

### 2.3. MicroRNAs Involved in the Response to HBV Treatment—A Bioinformatic Approach

The retrieved microRNAs from the first systematic review were included in a bioinformatic analysis using MIENTURNET software (http://userver.bio.uniroma1.it/apps/mienturnet, accessed on 2 May 2023) [34]. The purpose of this part of the study was to find the possible genetic connections between the microRNAs by assessing miRTarBase, following a Benjamini–Hochberg method. Then, we searched the potential interactions between the expression of these genes and their encoded proteins, using STRING software (https://string-db.org, accessed on 1 August 2023) [35]. We used this type of software because of its complexity: STRING (https://string-db.org, accessed on 1 August 2023) covers results from validated experiments, from software predictions, and data provided by a large number of online databases and by text-mining. The proteins encoded by the retrieved genes were identified using Ensembl database (https://www.ensembl.org/index.html, accessed on 2 May 2023) [36] and UniProt database (https://www.uniprot.org/, accessed on 2 May 2023) [37]. Our goal was to find the connections between the proteins influenced by microRNAs related to the response to HBV treatment.

### 2.4. The Known Effect of NUC or pegIFN over Lymphocytes in Chronic HBV Patients

Another step of our study was a systematic review conducted on 1897 studies. The search strategy included this time the following words and abbreviations (with their derivatives): “nucleoside”, “nucleotide”, “IFN (or interferon)”, “treatment”, “CHB (or chronic hepatitis B)”, “VHB”, “HBV”, “lymphocyte”, “leucocyte”, and “platelets”. The databases included for this research were PUBMED and Web of Science. All the retrieved results that were not articles were excluded, together with those without free and complete data. Articles written before 2013 were also not taken into consideration because we considered that the detection methods have evolved during the last decade. We also excluded articles not performed on humans. We only included articles that contained an observed effect of NUC or interferon-based therapies over the lymphocytes of chronic HBV subjects. The articles performed on fewer than 20 subjects were excluded because we only focused on studies with the best statistical power. Data of interest were related to general details about the study, to some of the characteristics of the included subjects (such as their number, their age, their ALT value, or their HBV-DNA levels), and to the general effect that therapy exerted over the lymphocytes. ZOTERO (http://www.zotero.org, accessed on 23 February 2023) [31] was used for data archives and the exclusion of duplicates. The PRISMA criteria [32] were also applied. The studies included were evaluated using Newcastle Ottawa Assessment Scale for Case–Control and Cohort studies [33]. The authors’ involvements were the same as those depicted in the previous review.

### 2.5. The Patient Selection Strategy 

The final study included 38 Romanian subjects with chronic HBV infection. Of these, 20 were being treated with pegIFN or NUCs for at least 6 months, while 18 of them did not receive any treatment during the entire duration of this research. The diagnosis of chronic HBV infection was previously established, so every patient presented, at enclosure, a positive HBsAg for more than six months. Both sexes were included. The selection of patients was carefully made, and the subjects diagnosed with other coinfections were eliminated from the lots. Pregnant women were also eliminated. Data acquisition was performed between 2020 and 2023, in Fundeni Clinical Institute in Bucharest, Romania. The patients gave written consent, and the approval of the Ethical Council of Fundeni Clinical Institute was obtained (number 46274). This article followed the Declaration of Helsinki. The research was performed on patients’ sera obtained from venous blood samples. On 23 of these patients (randomly selected) and another 5 healthy controls, we evaluated the expression of miR-122. The sera needed for the quantification of miR-122 were specially stored, at −80 °C, in a separate area for molecular testing. 

### 2.6. The Biochemical and Hemathological Parameter Acquisition

Biochemical data were assessed using Versacell V2 (Siemens Healthineers GmbH, Erlangen, Germany). Haemograms were obtained with SYSMEX-XN-1000-05 (Sysmex Europe, Norderstedt, Germany). ADVIA CENTAUR XPT_2 (Siemens Healthineers GmbH, Erlangen, Germany) was used for the detection of viral antigens, while the levels of viremia were detected with BOSPHORE VHB QUANTIFICATION KIT (Anatolia geneworks, Istanbul, Turkey). 

### 2.7. The Detection Method of miR-122 

MiR-122 was quantified according to a Thermo Fisher protocol. For this, 100 µL of sample serum was needed for each subject included in this part of the study. The first step consisted of the total RNA extraction. This was achieved after a digestion step with a buffer containing Proteinase K, followed by the lysis of the samples and by an RNA Binding method based on magnetic beads. The total RNA was finally eluted in 50 µL of Elution Buffer. After the extraction phase, reverse transcription was performed according to the manufacturer’s protocol (for each 15 µL of total reverse transcription reaction, we needed 10 ng of total RNA). The cycle of the reverse transcription phase was two periods of 30 min each at 16 °C, and then a period at 42 °C, followed by 5 min at 85 °C, and a cycle finish at 4 °C. The final phase of this microRNA detection method was the miRNA quantification with a real-time PCR amplification mode, which consisted of 1 cycle of 10 min at 95 °C for the activation of the enzyme, 40 cycles of denaturation at 95 °C (lasting 15 s), and an annealing/extension phase at 60 °C (lasting 60 s). A 2^−ΔΔCT^ method was used for establishing the relative expression of miR-122. The expressions of miR-122 were quantified using an internal control (which consisted of the expression of miR-21). Table 1 summarises the primers used in the reactions. The reagents used for miRNA quantification were provided by Applied Biosystems, Thermo Fisher Scientific, San Francisco, CA, USA. The quantification was made using Applied Biosystems 7300 (Applied Biosystems, Thermo Fisher Scientific, San Francisco, CA, USA).

### 2.8. The Methods Used for Statistical Analysis 

Statistical analysis using R 4.2.2 software (R Foundation for Statistical Computing, Vienna, Austria) provided the results for the final clinical study [38]. The normality in the distribution of the included variables was assessed using Shapiro’s test and the method of graphic representation. In the final analysis, we included the variables using medians or percentages (as needed). Univariate and multivariate analyses were performed. The Mann–Whitney test was used for intergroup differences (a *p*-value less than 0.05 was considered significant). After univariate analysis, Akaike Information Criteria were used to select the variables closely associated with treatment. The associations between the variables were evaluated with Factor Analysis for Mixed Data (FAMD), a method with a specific scaling system for retrieving the participation of the included variables at the dimensions of variability. The potential correlations were searched using Spearman’s test. The *p*-values were adjusted using a Holm method (they were considered significant below 0.05).

## 3. Results

### 3.1. A Systematic Review of the microRNA’s Involvement in the Response to HBV Treatment

**Figure 2:**
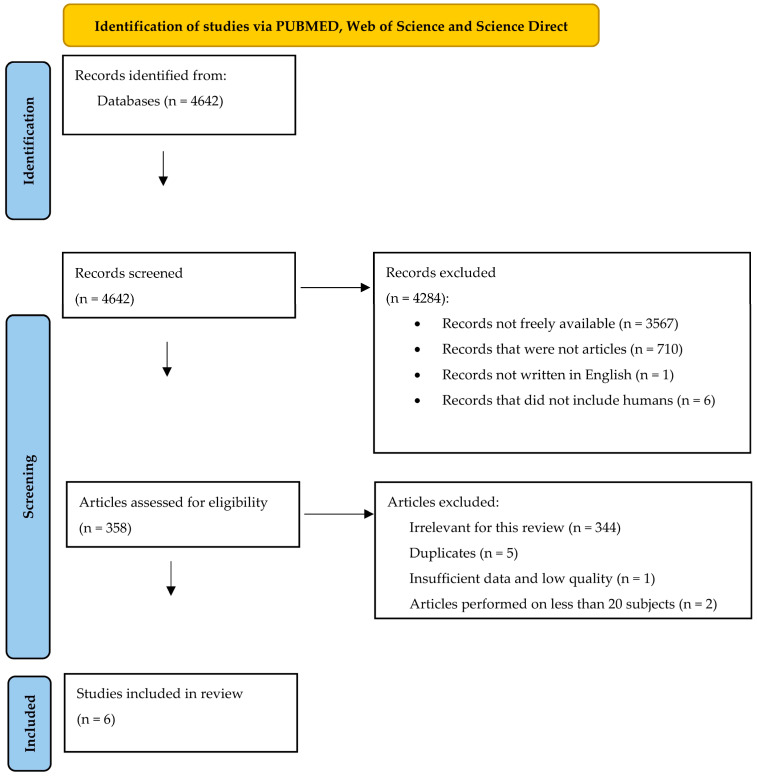
The selection diagram of the studies (adapted from the 2020 PRISMA Statement [32]).

After applying the research algorithm, we identified records from PUBMED (56 records), Web of Science (38 records), and Science Direct (4548 records). We then excluded 4284 records. Some of them were not freely available, others were not written between the 26 October 2023 and inception, while others were not written in English. We also excluded the records that were not articles, and the studies that were not performed on human subjects or human cell lines. Of the remaining 358 articles, we eliminated 5 duplicates, one study that did not contain enough relevant data, and 344 records that did not fit with the theme of this review. Studies performed on fewer than 20 patients were also eliminated. We finally analysed six articles. Our selection diagram is represented in Figure 2. Table 2 summarises the variables that we included in our analysis of the selected studies. We also mentioned the score of quality that we calculated for each study using the Newcastle Ottawa Assessment Scale for case-control and cohort studies [33]. 

The Newcastle Ottawa score was larger than 5 points in all the retrieved studies. The minimum of 6 points in this score was considered an acceptable level of quality. The main problem of the studies was that some of them [40,43,44] did not compare the values of the outcome (the studied microRNAs) at baseline and after the treatment period. Most of them did not provide proof for the studied outcome [40,41,42,43,44] (for example a table with the expression values of the microRNAs). Two studies [42,44] did not state clearly that the entire cohort from the beginning of the study was assessed till the end of the research. Concerning the results obtained, most of the studies were performed during the last six years [39,40,41,42], in Asian countries [39,40,41,42,44]. The entire systematic review summed up 387 patients, divided between treatment responders (134 subjects), and treatment nonresponders (253 subjects). As it was depicted in Table 2, treatment response was defined differently throughout the studies, which led to a high diversity of results. Despite this, one of our goals was to find a molecular relation between the retrieved articles, and we tried to do that in the following bioinformatic analysis (shown in Section 3.2). Regarding the method of analysis for the microRNA expression, we found that most studies used RT-PCR [39,40,42,43,44]. On the other hand, half of the articles presented the levels of microRNAs retrieved from the patients’ sera [39,40,41], while the other half used, for analysis, microRNAs found in plasma [42,43,44]. However, most microRNAs showed a higher expression in responders by comparison to nonresponders [39,41,43,44]. Most articles found the baseline moment of the study as the first moment of variation in the level of the microRNA, between the responders and the nonresponders [40,41,42,43,44]. 

### 3.2. Bioinformatic Analysis Based on the Findings of the First Systematic Review 

**Figure 3:**
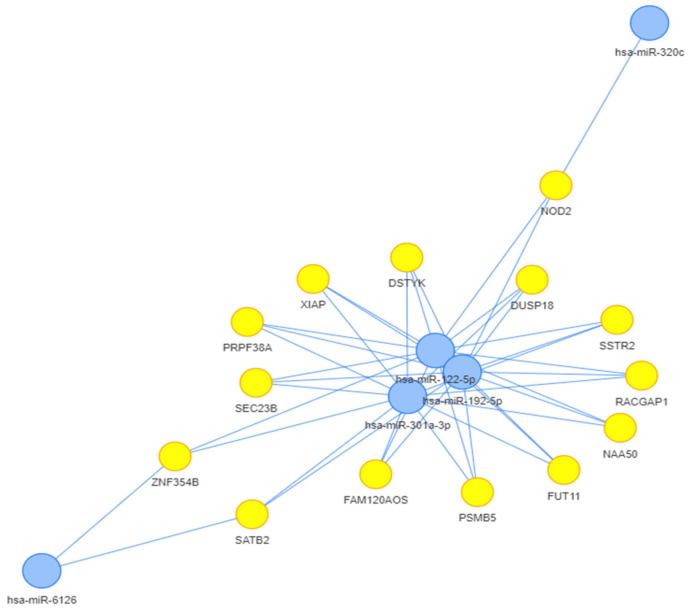
The microRNA-gene network relationship based on MIENTURNET analysis (http://userver.bio.uniroma1.it/apps/mienturnet, accessed on 2 May 2023) [34].

We performed a first bioinformatic analysis on the retrieved microRNAs from the first systematic review, using MIENTURNET software (http://userver.bio.uniroma1.it/apps/mienturnet, accessed on 2 May 2023) [34]. Only the microRNAs that were retrieved in miRTarBase, were included in this step of the study. The microRNAs of interest were considered those that were linked to each other with more than 3 connectors. The results from validated experiments mentioned in miRTarBase, from both strong and weak evidence, were included in this analysis, to obtain a broader view of the microRNA interactions. Thus, only 5 microRNAs remained as final focal points: miR-122-5p, miR-192-5p, miR-301-3p, miR-320c, and miR-6126. The connections between these microRNAs and the genes found by MIENTURNET (http://userver.bio.uniroma1.it/apps/mienturnet, accessed on 2 May 2023) are depicted in Figure 3.

**Figure 4:**
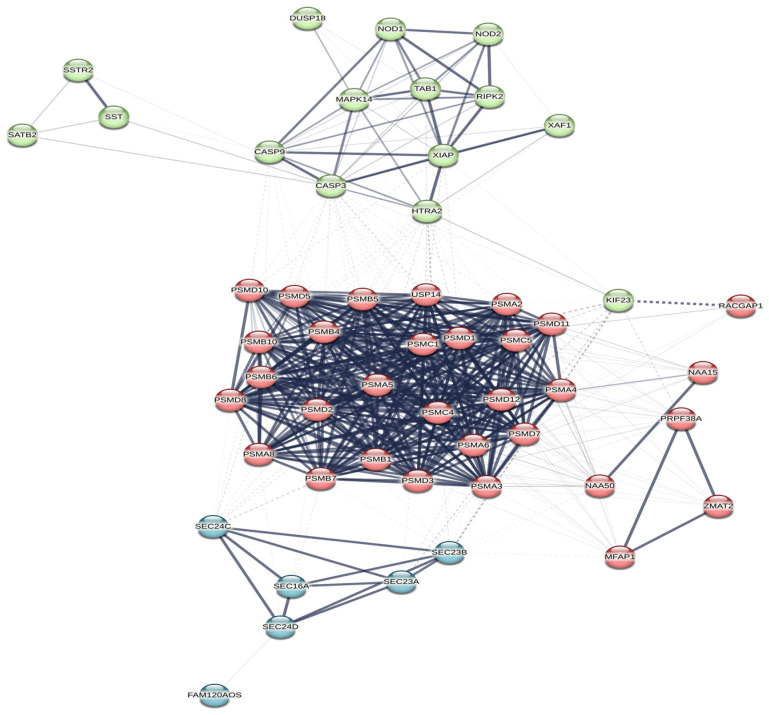
A STRING network (https://string-db.org, accessed on 1 August 2023) [35] depicting the relationship between the proteins encoded by the genes retrieved from MIENTURNET analysis (http://userver.bio.uniroma1.it/apps/mienturnet, accessed on 2 May 2023) [34]. The nodes coloured in the same colour belong to the same main cluster. The lines between the nodes show the high and the low connections between the proteins (high connections are shown with intense contoured lines, while low connections are shown with faint lines; high and low connections were established after the strength of the retrieved results). The dotted lines show the borders of the clusters that could be drawn from the connections between the proteins.

After the MIENTURNET analysis (http://userver.bio.uniroma1.it/apps/mienturnet, accessed on 2 May 2023) [34], we performed a second analysis on the STRING database (https://string-db.org, accessed on 1 August 2023) [35], using the names of the proteins encoded by the genes depicted in Figure 3. The names of these proteins were retrieved using Ensembl database (https://www.ensembl.org/index.html, accessed on 2 May 2023) [36], and UniProt (https://www.uniprot.org/, accessed on 2 May 2023) [37]. Most of these proteins were enriched in STRING (https://string-db.org, accessed on 1 August 2023) [35], thus we obtained a depiction of a statistically significant network (*p* < 0.001), which was shown in Figure 4. The network was designed automatically by the software, using a combined computed score method. Each protein received a score, according to the strength and the number of interactions in which it was involved. Three main clusters were discovered from the connections between the proteins. One of the clusters was linked to the activity of the proteasome (nodes are depicted in red in Figure 4), another one was related to the caspase and to the TRAIL (tumor necrosis factor related apoptosis inducing ligand [37]) activity (nodes are depicted in green in Figure 4), while the last main cluster was related to COPII (coat protein complex II [35]) vesicle transport (nodes are depicted in blue in Figure 4). Exploring the results provided by the STRING analysis (https://string-db.org, accessed on 1 August 2023) [35], we could find that the clusters with an FDR < 0.0001, and the highest strength (over 1.5) were related to the proteasome, the COPII vesicle coat, and the TRAIL signaling. STRING (https://string-db.org, accessed on 1 August 2023) [35] performed a functional enrichment analysis based on with information found in several databases such as Reactome (https://reactome.org/, accessed on 1 August 2023) [45], KEGG (https://www.genome.jp/kegg/, accessed on 1 August 2023) [46,47,48], Gene Ontology (https://geneontology.org/, accessed on 1 August 2023) [49,50], and WikiPathways (https://www.wikipathways.org/, accessed on 1 August 2023) [51]. KEGG (https://www.genome.jp/kegg/, accessed on 1 August 2023) [46,47,48] and WikiPathways (https://www.wikipathways.org/, accessed on 1 August 2023) [51] show a statistically significant connection (*p* < 0.05) between the STRING diagram (https://string-db.org, accessed on 1 August 2023) and both Hepatitis B and the TNF-alpha (tumor necrosis factor alpha [51]) pathway. Reactome (https://reactome.org/, accessed on 1 August 2023) [45] linked some of the proteins found in the STRING (https://string-db.org, accessed on 1 August 2023) diagram with the signaling cascades of the cytokines involved in the activation of the immune system. The pathways with the best strength (over 2.1) and the lowest FDR (*p* < 0.001), were related to ubiquitin and caspase regulation processes; NF-kB signals (including those related to B cell activity); NOTCH4, PTEN and RUNX3 activities; the degradation of AXIN, Emi1, NFE2L2 (nuclear factor erythroid 2-related factor 2 [37]), GLI2 (GLI family zinc finger 2 [37]), DVL and APOBEC3G. There was also a connection between some of the proteins retrieved and the adaptative immune system (1.18). 

### 3.3. A Systematic Review of Lymphocytes Affected by Antiviral Treatment in Chronic HBV Patients 

**Figure 5:**
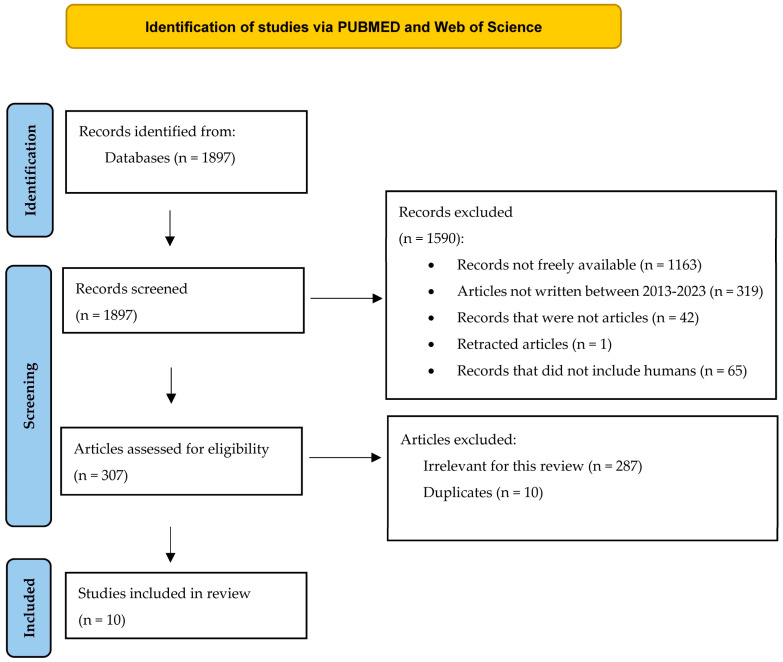
The selection diagram of the studies (adapted from the 2020 PRISMA Statement [32].

The selection algorithm, depicted in Figure 5, showed how we retrieved 1897 records from PUBMED and Web of Science. 1590 of these records were excluded, because of multiple reasons: 1163 of them were not freely available, 319 studies were not written between August, the tenth, 2023 and January, the first, 2013, and 42 records could not be included in the article category. 65 articles did not include human subjects or human cells, and 1 article was retracted by the authors. Then, 307 articles were assessed for eligibility, and 287 were found innappropriate for our systematic review (they did not fit the theme). 10 duplicates were also removed. The final analysis included 10 articles, and the retrieved data are summarised in Table 3. 

The search retrieved 10 studies performed in China between 2014 and 2023. We included in this systematic review only the articles with a quality score of over six points on the Newcastle–Ottawa scale, and we mentioned only the number of subjects who were used for the assessment of lymphocytes after HBV treatment. Three of the articles were case–control studies, encompassing 43 healthy control subjects versus 94 patients with chronic HBV infection [52,53,55]. Seven articles were cohort studies, gathering 405 patients found in various phases of chronic HBV infection [54,56,57,58,59,60,61]. The majority of the studies had a quality score of over seven. The most frequent elements which contributed to lowering the quality score were the incomplete depiction of the ascertainment of exposure [52,53,54,55,57,58] and of the nonresponse rate [54,55,57,58]. In one single article [61], immunohistochemical methods were used for the detection of lymphocytes in the liver biopsies taken from patients with chronic hepatitis B. The rest of the studies contained the description of the variations in the numbers of lymphocytes detected by flow cytometry [52,53,54,55,56,57,58,59,60]. Several articles showed a decrease in the numbers of T lymphocytes [52,55,61], starting from as early as week 8 of observation. Only two articles underlined the same decrease after NUC treatment [52,55]. PD-1 CD8+ T lymphocytes and T regulatory cells were both decreased at week 12 after NUC treatment [52], while pegIFN treatment seemed to decrease the numbers of CD8+ T lymphocytes in the livers affected by inflammation and fibrosis after week 24 of treatment [61]. However, HLA-DR^+^ CD 38^hi^ T lymphocytes were increased at week 12 of pegIFN treatment in patients with chronic HBV infection [59]. CD 95+ B lymphocytes were assessed in one single study, which found that these cells were increased in patients treated with NUCs after 12 weeks of therapy [53]. NK cells were also investigated in HBV patients. CD56^bright^NK cells were increased after 12 weeks [57,58] and 24 weeks [60] of therapy with NUCs and/or pegIFN. NK cells presented interest for the researchers in both the responder and the nonresponder groups selected after HBV treatment, and the studies showed that subjects with a good response after treatment also had an increase in NK cell numbers [54,56,57,58]. 

### 3.4. The Final Study—The Comparison between the Two Main Lots of Treated and Untreated Patients

The median age of the 38 patients (those receiving treatment and those without treatment) was 38.5 years, with unsignificant variations among the two categories of HBV-positive subjects. The subjects receiving treatment had been taking pegIFN or NUCs for at least 6 months. The dosage regimens were followed according to EASL (European Association for the Study of the Liver) guidelines [18]. The two lots also included patients with low viremia (under 2000 IU/mL) and high viremia (over 2000 IU/mL), with no significant differences between receiving treatment and those who did not receive it. However, the patients not receiving treatment had a significantly higher count of lymphocytes and platelets (with *p*-values under 0.05), in comparison with treated subjects. All subjects were HBsAg-positive. The results of these comparisons are included below, in Table 4.

### 3.5. Univariate and Multivariate Logistic Analyses Performed on Treated and Untreated Patients

The next step was the analysis of the associations between the variables presented above and the presence or absence of treatment. After the univariate logistic regression, only the counts of leucocytes, of lymphocytes, and of platelets were significantly associated with treatment in HBV-positive subjects (we considered significant, in univariate logistic analysis, *p*-values under 0.2 because multivariate analysis was used to confirm them; univariate analysis was used only as a trendsetter test). Both stepwise backward logistic regression and multivariate analyses eliminated the platelet count as a significant predictor of treatment in HBV-positive patients. Multivariate analysis showed that only the lymphocyte counts were changed significantly by HBV treatment. These results are depicted in Table 5 and Supplementary Table S1.

### 3.6. The Expression of MiR-122 

For 23 of the subjects selected from the lots above, we analysed the expression of miR-122 in comparison with a lot of 5 healthy controls. The median age of the control lot was 46 years, and it included 80% females and 20% males. All the subjects from the control lot were healthy, untreated volunteers. By comparison with the control lot, the expression of miR-122 was higher in patients receiving treatment (FC = 4.05), than that of the HBV positives not receiving treatment (FC = 2.42). The expression of miR-122 was stimulated in all the HBV-positive subjects. By analysing the results of the univariate analysis, we concluded that the expression of miR-122, together with the lymphocyte counts, were significantly modified in HBV patients receiving treatment, in comparison to those not receiving treatment (see Supplementary Table S2). We considered a *p*-value under 0.2 significant for the univariate analysis because such results were confirmed by the FAMD function in R 4.2.2 (R Foundation for Statistical Computing, Vienna, Austria) [38]. No significant correlation was found between the expression of miR-122 and the analysed biochemical or haematological parameters. We analysed the variables with a statistically significant variation caused by HBV treatment using the FAMD function in R 4.2.2 (R Foundation for Statistical Computing, Vienna, Austria) [38]. The results are displayed in Supplementary Figures S1 and S2. All the HBV-positive individuals were separated in different dimensions, according to the variability of their studied parameters. Only the first and the second dimensions were taken into consideration because they included most of the variations. The variations in the expression of miR-122 and the lymphocyte count were influenced, to a considerable extent, by therapy in patients receiving treatment. This suggests a possible association between the two. We have also added, in Supplementary Figure S3, the graphical representation of the expression of miR-122 in all the categories of subjects.

## 4. Discussion

MicroRNAs are currently considered molecules of interest for numerous diseases, due to their possible diagnostic and prognostic role. Some researchers even go further in giving potential therapeutic purposes to these noncoding particles. However, achieving the goal of treating future patients with microRNAs has not yet been established because of the lack of knowledge concerning the exact molecular mechanisms involved in human cells. To use microRNAs as treatment options, one must first find the exact noncoding molecules that target certain processes and obtain their antagonists. The latter should affect only those mechanisms that lead to the development of illness [62]. MiR-122 is one of the microRNAs intensely studied in chronically infected HBV patients. This noncoding molecule is stimulated in most chronic HBV patients, and it has been associated with the replication cycle of the HBV virus, together with pathways involving JAK/STAT (Janus kinase/signal transducer and activator of transcription), hepatocyte nuclear factor 4 (HNF4), and C/EBPα (CCAAT/enhancer-binding protein-β) [63]. However, the exact molecular signals that interfere with the functionality of miR-122 were not discovered yet. The impact of this microRNA on Romanian patients with chronic HBV infection is still a subject of debate. 

On the other hand, the effect of HBV on the human immune system is vastly studied [64,65] because of the potential treatment options targeted on immune receptors [66]. Studies have shown that HBV impairs both the innate and the acquired immune systems, leading to a decrease in NK cell number and activity, together with an increase in the number of regulatory lymphocytes (T reg). The persistence of HBV supports a continuous inflammation inside the human liver, thus causing an exhaustion of CD8+ T cells. The numbers and the functions of B cells are also affected, so all the premises of sustained immunosuppression are achieved. New immune therapies are currently under observation, and their purpose is to restore the normal functionality of the immune system [67]. Still, achieving immune therapies in HBV requires a better comprehension of the molecular activity of the regulators that act on lymphocytes and other immune cells.

In this study, we tried to evaluate the expression of miR-122 in chronic HBV patients by comparison with healthy subjects. The results showed a stimulation of miR-122 in all HBV patients, as compared to control subjects, thus following most of the previous findings in the literature, retrieved from non-Romanian subjects [63]. Another one of our obtained results was the observed relation between the lymphocyte numbers, the miR-122 levels, and the treatment status in Romanian patients with chronic HBV infection. This is the first study, as we know, that has such a result on a Romanian HBV patient lot. These results set a trend according to the bioinformatic analysis and the two systematic reviews presented in this article.

Therefore, our first systematic work was conceived to search the current knowledge comprising the microRNAs influenced by treatment response in chronic HBV patients. We chose as treatment options only pegIFN and NUC treatments because of their wide usage. Our research retrieved 10 microRNAs showing differences between treatment responders and nonresponders (miR-122 being one of them) [41]. More than half of the retrieved microRNAs had a higher expression in patients who responded to treatment in comparison to those who did not. Most of the observed differences in the expressions of these microRNAs were obtained at baseline measurements before treatment [41,42,43,44]. This suggests that some molecular factors interfere with microRNA functionality at a certain moment during HBV infection. These phenomena lead to the activation of several pathways with different results according to the patient’s infection status. With the addition of the effects generated by treatment, the result is that the expressions of certain microRNAs can be associated with the response in therapy. Finding the exact microRNAs involved in HBV pathogenesis might help researchers in predicting the response rate during HBV therapy or in changing treatment options. 

After the first systematic analysis, a bioinformatic analysis was performed on the genes influenced by the retrieved microRNAs. We then analysed the interactions between the proteins encoded by these genes. Half of the retrieved microRNAs (miR-122 included) could be linked together in a network. The final proteins (encoded by the genes influenced by the retrieved microRNAs) were also connected. Thus, we found a connection between miR-122-5p, miR-192-5p, miR-301a-3p, miR-6126, and miR-320c. By enriching the results and by using a bioinformatic analysis made by STRING (https://string-db.org, accessed on 1 August 2023) [35], we were able to discover the pathways in which these microRNAs were more likely involved together. 

Proteasome and ubiquitin signals were strongly connected with the proteins influenced mainly by miR-122-5p, miR-192-5p, and miR-301a-3p. Interestingly, the proteasome actions and the ubiquitin pathways are known to be related to immune cell activation [68,69]. On the other hand, molecules such as GLI 1, 2, and 3, APOBEC3G and DVL were related to proteasome–ubiquitin signals involving the retrieved microRNAs. GLI protein and its isoforms are zinc-based molecules with a role in the activation of gene transcription. APOBEC3G is a DNA deaminase with an observed antiviral action on HBV. DVL is a transferase with a documented role in cell signalling and differentiation [37]. Some cytokines (such as IL-2 and IL-5) were already identified as possible regulators of the APOBEC3G gene in lymphocytes [70]. IL-2 is also produced by impaired CD8+ T cells in chronic hepatitis B [71]. Other experiments show that GLI molecules and PI3K/AKT [72] (phosphatidylinositol-4,5-bisphosphate 3 kinase/AKT kinase [37]) influence lymphocyte survival and might trigger chronic lymphocytic leukaemia [72]. As such, a connection between some of the retrieved microRNAs from our first systematic analysis and lymphocyte proliferation and functionality might be encountered.

The activation of NF-kB signals in B cells is another pathway retrieved from the network shown in Figure 4. The main proteins related to this pathway are mainly the ones influenced by miR-122-5p, miR-192-5p, and miR-301a-3p. Studies have previously shown that NF-kB signals are also associated with monocyte and NK cell suppression in HBV infection [73]. However, NF-kB is responsible for B cell differentiation and activation, being one of the key signalling points that connect T lymphocytes with B lymphocytes [74]. HBV is known to block NF-kB signals and, thus, to achieve a low innate IFN (interferon) type I secretion [65]. Our retrieved network (Figure 4) also identifies a regulator pathway for NF-kB, in which nucleotide-binding oligomerisation domain-containing protein 2 (NOD2) is involved. NOD2 is a protein that regulates MAPK (Mitogen-activated protein kinase) signals and interferes with the immune control exerted by Tregs and T helper lymphocytes [37]. The main microRNA which influences NOD2 is miR-320c, according to our bioinformatic analysis.

Apoptotic pathways were also activated mainly by the proteins regulated by miR-6126, miR-320c, miR-122-5p, miR-192-5p, and miR-301a-3p. These proteins are DUSP (dual specificity protein phosphatase [37]), XIAP (X-linked inhibitor of apoptosis [37]), SATB2, SSTR2 (somatostatin receptor type 2 [37]), and NOD2. DUSP was studied before as a regulator of T cell activity in autoimmune illnesses [75] and as an important element of the MAPK activity in lymphocyte proliferation [76]. On the other hand, XIAP is also a regulator of CD8+ T cell proliferation [77]. SATB2 is connected to other protein kinases in the process of tumorigenic transformation of B cells, through BCR–ABL [78]. Other studies describe SSTR2 as an immunologic receptor placed on various cells, which regulates T cell functionality [79,80]. 

To sum up, the connections found in the network from our bioinformatic analysis (Figure 4) are linked to the activity and the proliferation of the immune cells, mostly of lymphocytes. MiR-122 is one of the microRNAs with a constant involvement in all these inter-relations, as is depicted in Figure 3 and Figure 4. MIENTURNET (http://userver.bio.uniroma1.it/apps/mienturnet, accessed on 2 May 2023) [34] and STRING (https://string-db.org, accessed on 1 August 2023) [35] helped us achieve a series of molecular connections between miR-122 and other microRNAs, which had different expressions in treatment responders and nonresponders. 

Encouraged by our findings from the first part of our study and from our bioinformatic analysis, we performed a second systematic review. Since the microRNAs related to the response to HBV treatment were linked to lymphocytes, we searched for a deeper connection between treatment and the levels of lymphocytes. Therefore, the second systematic review was centred on the effect of HBV therapy (NUCs and pegIFN) on the number of lymphocytes.

The lymphocyte counts were also influenced by the response to therapy in various ways [52,53,54,55,56,57,58,59,60,61]. Studies showed that both NUCs and pegIFN help in restoring the numbers of NK cells [56,57,58,60]. The experiments involving NUC treatment proved that this therapy helps in suppressing T regs [52,55] and in restoring B cells [53]. However, the results of pegIFN on the numbers of CD8+ T cells were not good in patients with advanced stages of fibrosis and inflammation [61]. The molecular basis for all these results was not clearly stated in any of the retrieved studies. In contrast to the differences in the expressions of microRNAs, the significant increase or decrease in the numbers of the lymphocytes was later observed during treatment (mostly on week 12 and on week 24) [52,53,54,55,56,57,58,59,60,61]. The second systematic analysis retrieved several types of lymphocytes that were influenced by treatment or that were related by themselves to the treatment course. The same categories of lymphocytes could be influenced by the microRNAs included in our bioinformatic analysis. 

Our final hypothesis was that several microRNAs (miR-122 included) could determine the proliferation or the activity of lymphocytes, thus contributing to the events involved in treatment response and nonresponse. That is why we concluded our research with a study performed on miR-122 and chronic HBV patients receiving treatment. At this point, we first proved a clear influence that HBV treatment has on the counts of lymphocytes. The levels of lymphocytes in the patients’ sera were lower in patients receiving treatment as compared to those who were not receiving treatment. These results obtained on Romanian patients are like others found in the second systematic review [52,55]. Then, we showed that, in patients receiving therapy for a chronic HBV infection the variations between the levels of miR-122 and the lymphocyte counts were closely related. This is an argument that supports our previous theories and findings. The interaction between miR-122 and lymphocytes is enhanced by HBV therapy. This is a new molecular explanation that sets a trend towards the link between miR-122 and the levels of lymphocytes previously shown in the first bioinformatic analysis. 

The major limitations of this study are related to the relatively small lot of chronically infected HBV patients that we took into consideration in the last clinical study. Another potential limitation of this article might be related to the fact that we did not assess the evolution in time of the expression of miR-122 in patients receiving treatment and those not receiving treatment. 

However, our study has an interesting applicability. It sets a trend, for the first time, towards the connection between miR-122 and lymphocytes in Romanian patients receiving treatment. Although both miR-122 and the levels of lymphocytes are related to the response to therapy, these connections are not fully understood, and they might be different from what has been discovered until now. This might be because of the differences in the viral genotypes encountered in Romanians as opposed to the other populations already studied [29,30] since HBV genotypes are important in the response to therapy [28]. Therefore, our study sets a trend towards future research projects which have the potential to better define the effects of HBV treatment on all individuals at a molecular level.

The novelty of our research resides both in its complexity and its theme, and it might be a step forward in deciphering the molecular interactions caused by HBV treatment. This could help clinicians in giving their patients personalised treatment, with a better response rate and better survival.

## 5. Conclusions

The problem of microRNAs and their mechanisms of genetic regulation is still under debate. This study provides a new perspective in the understanding of the molecular pathways in HBV patients, by presenting the intricate and complex relations between miR-122 and other microRNAs and by suggesting a link between miR-122 and lymphocytes. Our article is the first one following this subject performed on Romanian patients, and it might encourage future discoveries on a better prediction of the response to treatment in chronic HBV infection.

## Figures and Tables

**Table 1:** The used primers. The primer sequences were complementary to the sequences provided below.

Assay	Assay ID/Catalog Number	Mature miRNA Sequence	Chromosome Location
hsa-miR-21	000397/4427975	UAGCUUAUCAGACUGAUGUUGA	Chr.17
hsa-miR-122	002245/4427975	UGGAGUGUGACAAUGGUGUUUG	Chr.18

**Table 2:** The main data of the included studies, which presented microRNA values related to the response to HBV treatment.

First Author; Reference	Year	Study Location	Study Design	Sample Size	Detection Method for microRNA	Expression of microRNA	Treatment	Moment When the Outcome Was First Observed (from the Beginning of Study)	NOS *
Fujita, K. [39]	2018	Japan	Cohort and cell-culture	6 treatment responders versus 16 nonresponders (treatment responders had a 1-log decrease in the level of HBsAg as compared to baseline)	Microarray, RT-PCR *	MiR-6126-higher in responder’s sera	PegIFN+/−NUC	Week 24	9
Nagura, Y. [40]	2022	Japan	Cohort	12 treatment responders versus 49 nonresponders (treatment response was established based on ALT < 31 U/L, HBV-DNA < 2000 IU/mL, on the absence of NUCs till week 48, and on the absence of HBeAg)	RT-PCR *	MiR-192-5p-lower in responder’s sera	PegIFN	Baseline (under no treatment)	7
Tan, B. [41]	2021	China	Cohort	18 treatment responders versus 18 nonresponders (treatment response was based on the absence of HBeAg)	Sequencing	MiR-122-5p- lower in responder’s sera; MiR-1307-3p, miR-320a-3p, miR-320c-higher in responder’s sera	Entecavir+ PegIFN	Baseline (under no treatment) for miR-122-5p and miR-1307-3p; Week 48 for miR-320a-3p and miR-320c	8
Ouyang, Y. [42]	2019	China	Cohort	28 treatment responders versus 87 nonresponders (treatment response was based on the absence of HBeAg)	RT-PCR *	MiR-146a-lower in responder’s plasma	NUCs	Baseline (under no treatment)	7
van der Ree, M.H. [43]	2021	Netherlands	Cohort and cell culture	14 treatment responders versus 27 nonresponders (treatment response was based on the absence of HBeAg, normal ALT levels, and HBV-DNA < 2000 IU/mL)	RT-PCR *	MiR-301-3p-higher in responder’s plasma	PegIFN +NUCs	Baseline (under no treatment)	7
Li, J. [44]	2017	China	Cohort	56 treatment responders versus 56 nonresponders (treatment response was based on an early decrease by 2 log10 of the HBV-DNA, and then sustained with a viremia less than 2000 IU/mL and the absence of HBeAg)	RT-PCR *	MiR-22 and miR-210-higher in responder’s plasma	Interferon based	Baseline (under no treatment)	6

* RT-PCR-real-time PCR, NOS- Newcastle Ottawa Assessment Scale for case control and cohort studies.

**Table 3:** The main data of the included studies, which presented the categories of lymphocytes influenced by HBV treatment.

First Author; Reference	Year	Study Location	Study Design	Sample Size	Detection Method for Lymphocytes	Outcome (Treatment Effect on Lymphocytes)	Treatment	Moment When the Outcome Was First Observed (from the Beginning of Study)	NOS
Li, C.-Z. [52]	2014	China	Case control	14 healthy controls versus 52 HBV patients with chronic hepatitis, divided in 2 equal groups (one group of lamivudine treated subjects and another one of telbivudine treated subjects)	Flow Cytometry	A decrease in time of the number of PD-1 CD8+ T cells and of T regulatory cells	NUCs *	Week 12	8
Zhao, P.-W. [53]	2015	China	Case control	17 healthy subjects versus 15 patients with chronic HBV infection, treated with adefovir dipidoxil	Flow Cytometry	An increase in time in the number of CD 95+ B cells	NUCs *	Week 12	8
Cao, W. [54]	2021	China	Cohort	89 patients with chronic hepatitis B (49 subjects treated with pegIFN versus 40 subjects treated with Entecavir- both groups were divided in treatment responders versus nonresponders; responders were established at 48 weeks among those with a decrease in HBsAg of >60% in the pegIFN group, and those with an undetectable level of HBV-DNA in the Entecavir group)	Flow Cytometry	An increase in time in the frequency of NK * cells in both pegIFN and Entecavir groups; The NK * cell frequency was increased both in the responder group of pegIFN treated subjects, and in the nonresponder group of Entecavir subjects.	Entecavir/pegIFN	Week 12	7
Yang, X. [55]	2017	China	Case control	12 healthy controls versus 27 chronic hepatitis B patients, treated with lamivudine	Flow Cytometry	A decrease in time in the number of T cells	NUCs *	Week 8	7
Shen, X. [56]	2016	China	Cohort	92 patients with chronic hepatitis B, treated with pegIFN or pegIFN+ adefovir dipivoxil (they were separated in 2 groups—17 responders versus 75 nonresponders; treatment response was established at the end of the follow-up, and it was defined as the loss of HBeAg and HBV-DNA < 2000 IU/mL)	Flow Cytometry	An increase in time in the NK * cell number in responders versus nonresponders	pegIFN+/− NUCs	Week 12	9
Chen, T. [57]	2017	China	Cohort	52 patients with chronic hepatitis B (divided in several groups with different ALT, HBV-DNA values, or with HBeAg present or absent)	Flow cytometry	An increase in NKG2D^+^ CD56^bright^ NK * cells in patients with HBeAg seroconversion at 36 weeks; the percentage of NK * cells was increased in subjects with high levels of HBV-DNA at 12 weeks	NUCs *	Week 12 for the difference in the NK * cells’ percentage; week 36 for the increase in NKG2D^+^ CD56^bright^ NK * cells	8
Cao, W. [58]	2022	China	Cohort	66 patients with chronic hepatitis B (divided in 2 groups: 17 subjects achieved functional cure and 49 patients were included in a nonfunctional cure category; the follow-up was then performed on 14 patients, and on 40 subjects; functional cure was defined as the absence of HBsAg and HBeAg, together with the undetectable level of HBV-DNA)	Flow cytometry	An increase in the percentage of CD56^bright^ NK * cells/NK * cells in patients with functional cure	pegIFN+/− NUCs *	Week 12	7
Lin, Y. [59]	2023	China	Cohort	34 patients treated only with pegIFN; 10 patients treated with pegIFN and NUCs *, and 15 patients with intermittent pegIFN treatment	Flow cytometry	An increase in time in the HLA-DR^+^ CD 38^hi^ T lymphocytes in long term treatment with pegIFN	pegIFN+/− NUCs *	Week 12	8
Pang, X. [60]	2020	China	Cohort	40 patients (divided in 2 groups—33 subjects treated with NUCs *, and 7 subjects treated with pegIFN+ NUCs *)	Flow cytometry	A persistent decrease in the number of CD56^dim^ NK * cells in patients treated with pegIFN and NUCs *; a persistent increase in the number of CD56^bright^NK * cells in patients treated with pegIFN and NUCs	NUCs *+/− pegIFN	Week 24	9
Liu, R. [61]	2020	China	Cohort	32 patients with chronic hepatitis B (divided into several groups based on their inflammation–fibrosis scores on liver histology)	IHC * staining	A decrease in CD8^+^ T lymphocytes in the persistent fibrosis-inflammation group	pegIFN	Week 24	9

* NK—natural killer, NUC—nucleotide/nucleoside analogues, IHC—immunohistochemical.

**Table 4:** Clinical comparisons between the two main lots of patients.

Variable	Total (n = 38)	Receiving Treatment (n = 20)	Not Receiving Treatment (n = 18)	*p*-Value **
Age (years)	38.5 [32.2, 53]	36.5 [32.8, 57.8]	40.5 [32.5, 52]	0.988
Sex—female (%)	16 (42.1)	7 (35)	9 (50)	0.544
ALT * (U/L)	26.5 [21.2, 41.5]	27 [23.5, 47.2]	26.5 [21, 37]	0.715
AST * (U/L)	25.5 [23, 35]	28.5 [22.8, 36]	25 [23, 26.8]	0.319
Total Bilirubin (mg/dL)	0.6 [0.5, 0.8]	0.6 [0.6, 0.8]	0.6 [0.5, 0.8]	0.767
PT * (seconds)	14 [13.2, 14.7]	14.2 [13.6, 14.9]	13.6 [13, 14.7]	0.224
Leucocyte count (×10^3^ µL)	7.1 [5.9, 8.4]	6.3 [5.8, 7.6]	7.6 [6.4, 9.6]	0.067
Lymphocyte count (×10^3^ µL)	2.2 [1.7, 2.4]	1.9 [1.6, 2.2]	2.3 [1.9, 2.5]	0.023
Platelet count (×10^3^ µL)	262 [215, 307.5]	224 [204.8, 271.5]	298.5 [260.8, 328.8]	0.005
HBV-DNA (IU/mL)	2514 [326, 14,323.8]	1635 [66.8, 7932.8]	4228 [2045, 17,826.2]	0.128

* ALT—alanine aminotransferase, AST—aspartate aminotransferase, PT—prothrombin time; ** *p*-value < 0.05 was considered significant.

**Table 5:** The results of univariate logistic analysis.

Variables	OR *	95% CI *	*p*-Value **
Age (years)	1	0.96, 1.05	>0.9
Sex—male (%)	1.86	0.51, 7.07	0.4
ALT * (U/L)	1	0.99, 1	0.7
AST * (U/L)	1	0.99, 1.01	0.7
Total Bilirubin (mg/dL)	0.89	0.47, 1.25	0.5
PT * (seconds)	1.43	0.86, 2.58	0.2
Leucocyte count (×10^3^ µL)	0.73	0.5, 1	0.068
Lymphocyte count (×10^3^ µL)	0.16	0.03, 0.63	0.02
Platelet count (×10^3^ µL)	0.99	0.98, 1	0.075
HBV-DNA (IU/mL)	1	1, 1	0.5

* OR—odds ratio, CI—confidence interval, ALT—alanine aminotransferase, AST—aspartate aminotransferase, PT—prothrombin time; ** *p* < 0.2 was considered significant.

## Data Availability

Not applicable.

## Supplementary Materials

The following supporting information can be downloaded at: https://www.mdpi.com/article/10.3390/microorganisms11112731/s1, Figure S1. FAMD results for treated patients; Figure S2. The contribution of miR-122 and lymphocytes in treated patients; Figure S3. Different expression of miR-122 among the included categories; Table S1. The statistically significant models included in multivariate analysis; Table S2. The expression of miR-122 in univariate logistic analysis.
