# Supplementary material for: The Connection between MiR-122 and Lymphocytes in Patients Receiving Treatment for Chronic Hepatitis B Virus Infection
Source: Microorganisms. 2023 Nov 8;11(11):2731. doi: 10.3390/microorganisms11112731 (PMC10673475; doi:10.3390/microorganisms11112731)
Supplement: Supplementary file 1 [file microorganisms-11-02731-s001.zip › Figure S1.pdf]

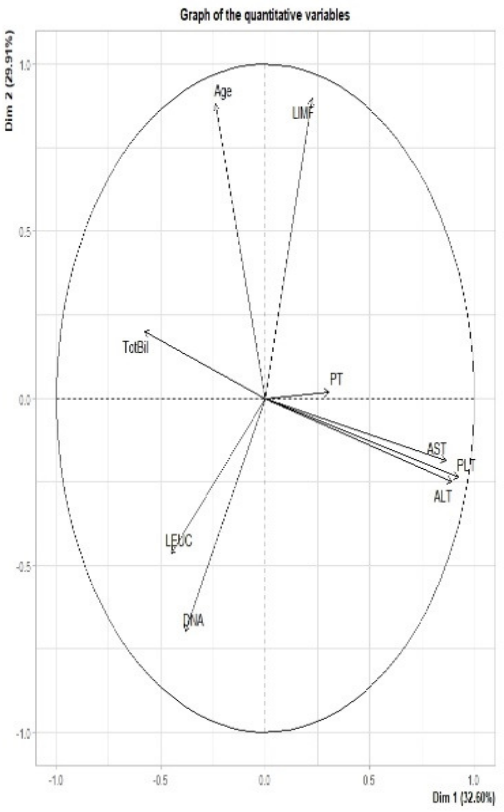

(**a**)

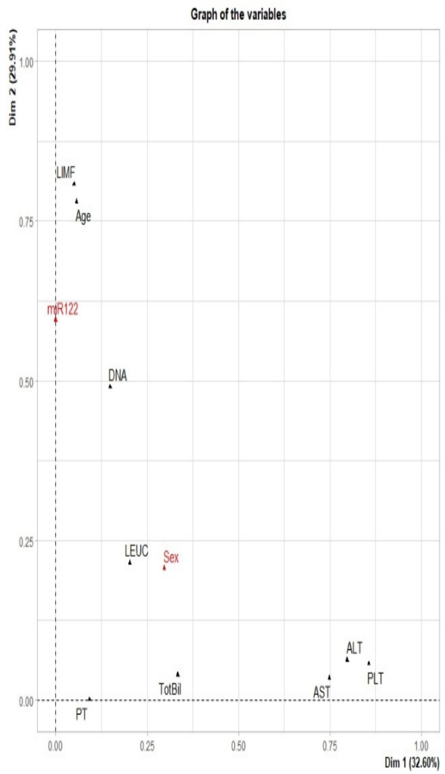

(**b**)

Figure S1. FAMD results for patients receiving treatment. (**a**) The FAMD variations of the main variables significantly modified by miR-122; (**b**) The expression of miR-122 and the lymphocyte counts were closely associated. ALT-alanine aminotransferase; AST-aspartate aminotransferase; TotBil- total bilirubin; DNA- the value of HBV DNA; PT-prothrombin time; PLT-platelet count; LIMF-lymphocyte count; LEUC-leucocyte count.
