# Supplementary material for: The Connection between MiR-122 and Lymphocytes in Patients Receiving Treatment for Chronic Hepatitis B Virus Infection
Source: Microorganisms. 2023 Nov 8;11(11):2731. doi: 10.3390/microorganisms11112731 (PMC10673475; doi:10.3390/microorganisms11112731)
Supplement: Supplementary file 1 [file microorganisms-11-02731-s001.zip › Figure S2.pdf]

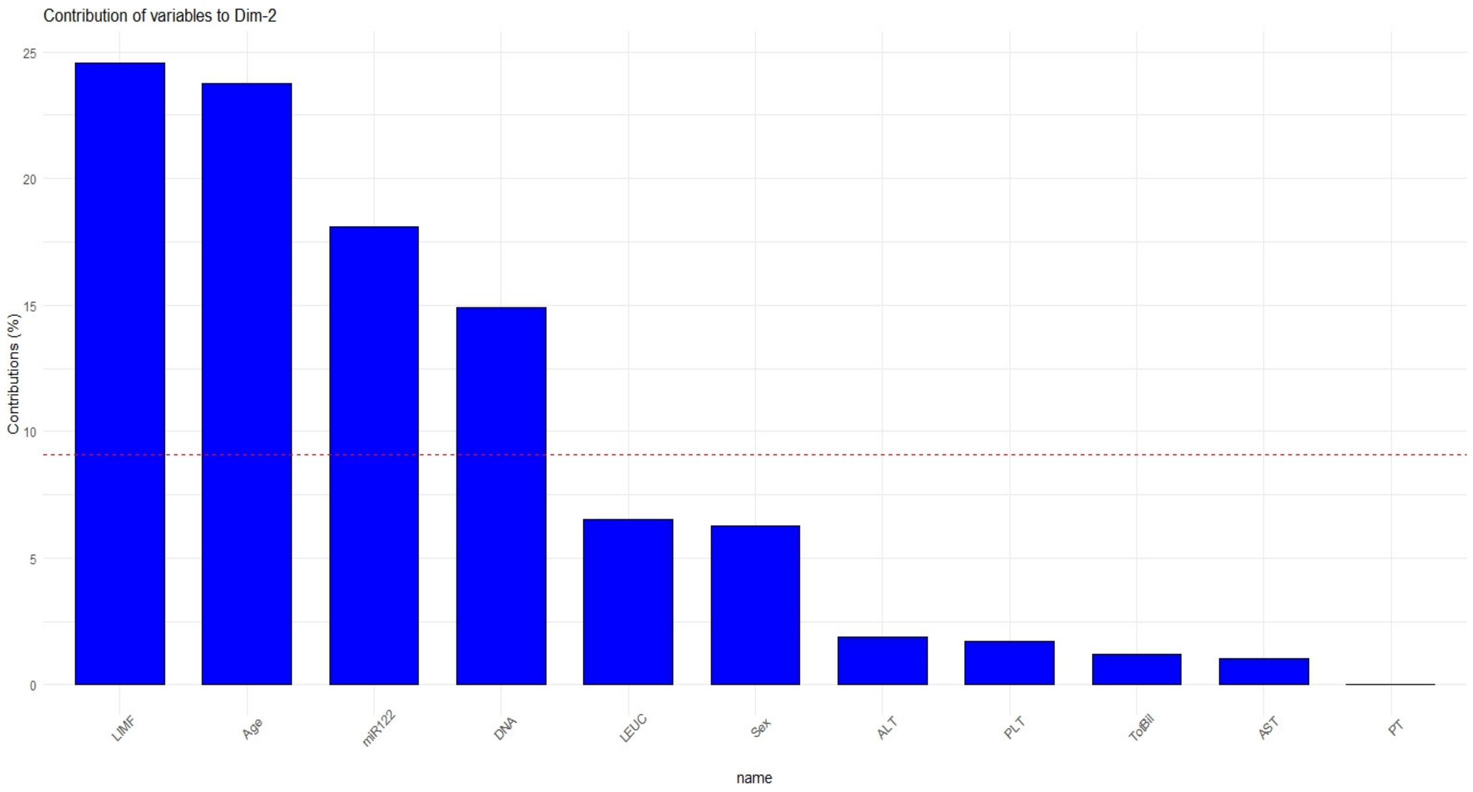

Figure S2. The contribution of miR-122 and lymphocytes in patients receiving treatment. The dominant contribution of the expression of miR-122 and the lymphocyte count to the FAMD dimension (dimension 2). LIMF-lymphocyte count; LEUC-leucocyte count; ALT-alanine aminotransferase; AST-aspartate aminotransferase; TotBil-total bilirubin; DNA-HBV-DNA value; PT-prothrombin time; PLT-platelet count.
