# Supplementary material for: The Connection between MiR-122 and Lymphocytes in Patients Receiving Treatment for Chronic Hepatitis B Virus Infection
Source: Microorganisms. 2023 Nov 8;11(11):2731. doi: 10.3390/microorganisms11112731 (PMC10673475; doi:10.3390/microorganisms11112731)
Supplement: Supplementary file 1 [file microorganisms-11-02731-s001.zip › Figure S3.pdf]

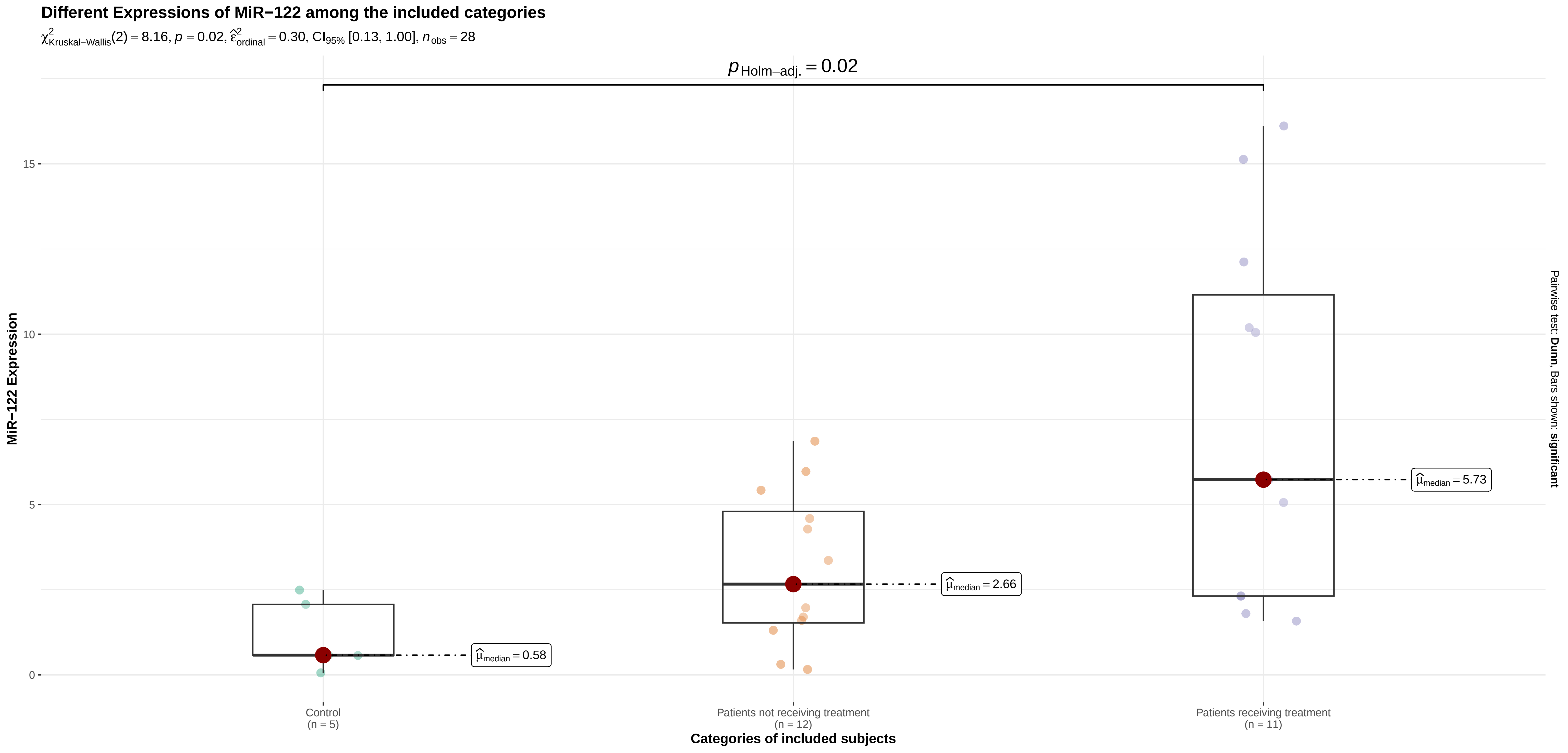

Different Expressions of MiR−122 among the included categories
$\chi^2_{\text{Kruskal-Wallis}}(2) = 8.16$, $p = 0.02$, $\hat{\varepsilon}^2_{\text{ordinal}} = 0.30$, $\text{CI}_{95\%}$ [0.13, 1.00], $n_{\text{obs}} = 28$
$p_{\text{Holm-adj.}} = 0.02$
$\hat{\mu}_{\text{median}} = 0.58$
$\hat{\mu}_{\text{median}} = 2.66$
$\hat{\mu}_{\text{median}} = 5.73$
MiR−122 Expression
0
5
10
15
Control
(n = 5)
Patients not receiving treatment
(n = 12)
Patients receiving treatment
(n = 11)
Categories of included subjects
Pairwise test: Dunn, Bars shown: significant
