# Supplementary material for: The Connection between MiR-122 and Lymphocytes in Patients Receiving Treatment for Chronic Hepatitis B Virus Infection
Source: Microorganisms. 2023 Nov 8;11(11):2731. doi: 10.3390/microorganisms11112731 (PMC10673475; doi:10.3390/microorganisms11112731)
Supplement: Supplementary file 1 [file microorganisms-11-02731-s001.zip › Table S1.pdf]

Table S1. The statistically significant models included in multivariate analysis.

	Model 1			Model 2		
**Estimates**	**Estimates**	**95% CI***	***p*-value****	**Estimates**	**95% CI***	***p*-value****
(Intercept)	1.07	0.51-1.63	<0.001	1.27	0.72-1.82	<0.001
Leucocyte counts ($x10^3$µl)	-0.07	-0.15-0	0.05			
Lymphocyte counts ($x10^3$µl)				-0.36	-0.62--0.11	0.006

* CI-confidence interval; ***p* < 0.2 was considered significant.
