# Supplementary material for: The Connection between MiR-122 and Lymphocytes in Patients Receiving Treatment for Chronic Hepatitis B Virus Infection
Source: Microorganisms. 2023 Nov 8;11(11):2731. doi: 10.3390/microorganisms11112731 (PMC10673475; doi:10.3390/microorganisms11112731)
Supplement: Supplementary file 1 [file microorganisms-11-02731-s001.zip › Table S2.pdf]

Table S2. The expression of miR-122 in univariate logistic analysis.

Variables	OR*	95% CI*	*p*-value**
Age (years)	1	0.9, 1.4	0.4
Sex-male (%)	0.6	0.11, 3.09	0.5
ALT* (U/l)	1	0.98, 1	0.5
AST* (U/l)	1	0.96, 1.01	0.5
Total Bilirubin (mg/dl)	0.82	-	0.5
PT* (seconds)	1.39	0.7, 3	0.4
Leucocyte count (x10³µl)	0.76	0.47, 1.11	0.2
Lymphocyte count (x10³µl)	0.28	0.04, 1.21	0.13
Platelet count (x10³µl)	0.99	0.98, 1	0.2
HBV-DNA (IU/ml)	1	1, 1	0.5
miR-122	1.34	1.06, 1.93	0.047

*OR- odds ratio, CI- confidence interval, ALT- alanine aminotransferase, AST- aspartate aminotransferase, PT- prothrombin time; ***p* < 0.2 was considered significant.
